# Homologous Recombination Deficiency Testing in Women With Ovarian Cancer: An Egyptian Multicentre Study

**DOI:** 10.1111/1471-0528.70052

**Published:** 2025-10-06

**Authors:** Kyrillus S. Shohdy, Loay Kassem, Boules Gabriel, Emad Barsoum, Tamer Elnahas, Hamdy A. Azim

**Affiliations:** ^1^ Department of Clinical Oncology Cairo University Cairo Egypt; ^2^ School of Cancer Sciences and CRUK Scotland Institute University of Glasgow Glasgow UK; ^3^ Department of Medical Oncology Nasser Institute for Research and Treatment Cairo Egypt; ^4^ Barsoum Oncology Center Cairo Egypt

Identification of patients with homologous recombination deficiency (HRD) is crucial for the better management of patients with epithelial ovarian cancer (EOC) [1]. In limited‐resource countries, there is a shortage of HRD testing outcomes due to costly testing, substandard infrastructure and lack of expert know‐how [2].

We conducted a multicentre registry study aiming to evaluate the feasibility and outcomes of in‐house HRD testing for patients with EOC in Egypt (Figure 1A). From 2019 to 2022, 589 consecutive patients with a median age of 56 years (range 20–86), from five cancer centres across Egypt, had undergone a successful HRD testing. The study was approved by the Institutional Review Board at Dar El Salam Cancer Centre (Ministry of Health and Population—Egypt).

**FIGURE 1:**
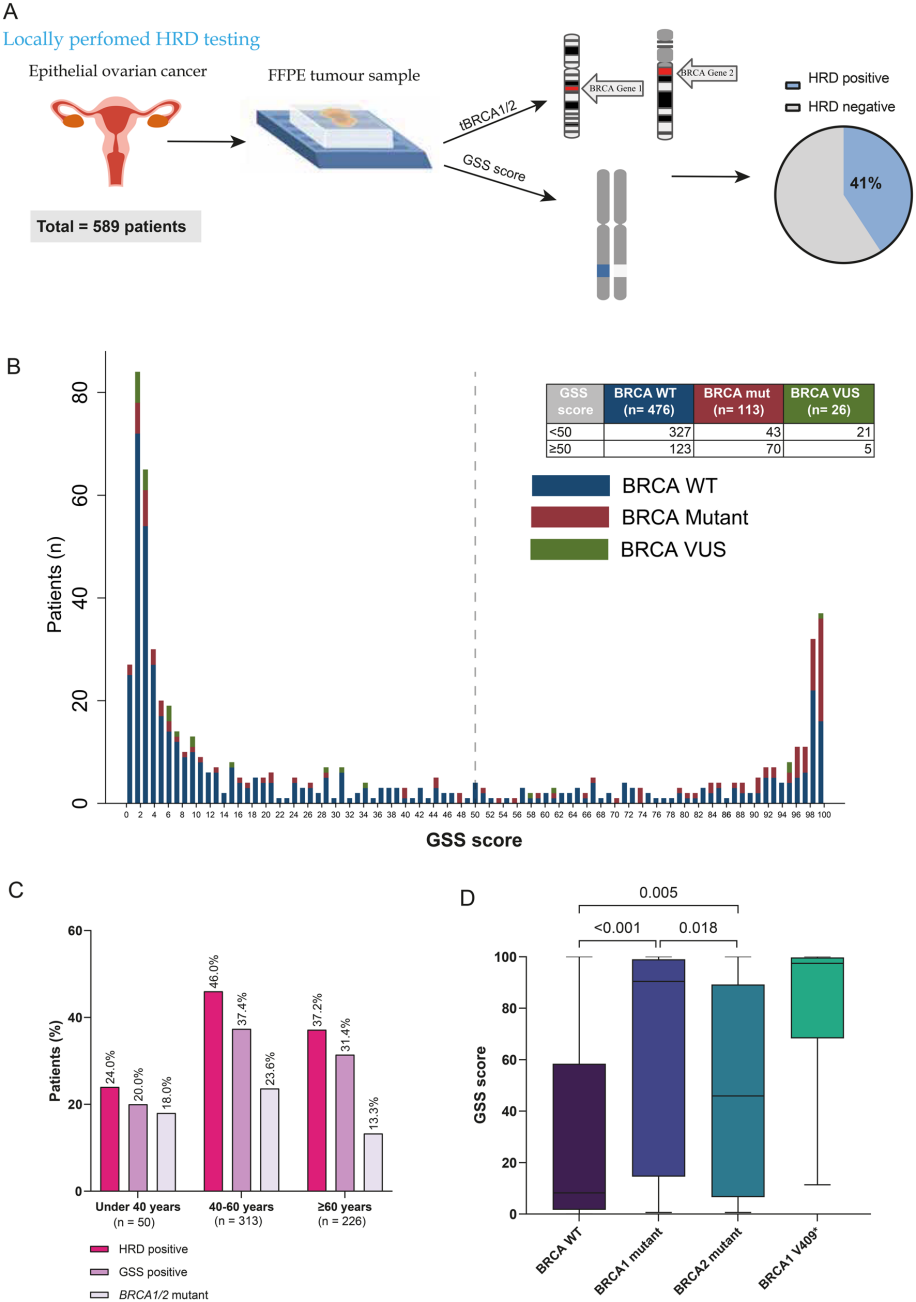
(A) Schematic overview of the study design and outcomes. (B) Distribution of genomic scare score (GSS) across all patients in relation to the *BRCA1/2* mutation status, wild type, mutant and VUS. (C) Barplots showing the frequencies of patients with HRD and GSS score‐positive and *BRCA* mutation across the age categories (under 40 years, 40 to < 60 years and ≥ 60 years). (D) Boxplots showing the GSS score in patients with BRCA1/2 wild‐type in comparison with *BRCA1 and BRCA2* mutant patients, middle horizontal line is the median and the whiskers represent min and max data points.

HRD testing included *BRCA1/2* tumour mutation status (t*BRCA1/2*) and genomic scar score (GSS) status. Briefly, for each FFPE sample submitted, DNA extraction was performed using QIAamp DNA Mini kit (QIAGEN). Then, appropriate libraries preparation according to the manufacturer's protocol with the AmoyDx HRD Panel (Amoy Diagnostics Co. Ltd.) and sequencing was performed using the Illumina NextSeq500 system (Illumina). A custom hybridisation capture panel that targets 27 000 SNPs distributed across the genome was used to infer the GSS. Patients with *BRCA1/2* mutations or GSS ≥ 50 (i.e., GSS‐positive) were considered HRD‐positive as previously validated [3] (Figure 1A).

GSS‐positive tumours were detected in 198 (33.6%) of our patients while t*BRCA1/2* mutations were detected in 113 (19.2%) (Figure 1B). Collectively, 241 patients (41%) were diagnosed as HRD‐positive EOC at a median turnaround time of 13 days (range 8–26 days).

We examined the contribution of the GSS score and t*BRCA1/2* status to the overall HRD positivity (Figure 1B). In total, 128 patients (21.7%) were positive due to a positive GSS score alone without identified t*BRCA1/2* mutations. Meanwhile, 43 (7.3%) were HRD‐positive due to having *BRCA1/2* mutations with a negative GSS score. Expectedly, the majority (62%) of patients with *BRCA* mutations had a positive GSS score, and the majority (80.7%) of patients with *BRCA* variants of uncertain significance (VUS) were GSS‐negative (Figure 1B).

There was no significant difference in HRD‐positive rate in younger age (< 50‐year‐old) compared to older (63 (38.2%) vs. 178 (42%), *p* = 0.42) neither in the rate of GSS‐positive (51 (30.9%) vs. 147 (34.7%), *p* = 0.38). However, we identified a significant trend for increasing the rate of HRD‐positive across age categories with the highest at age category 40 to < 60 years and lowest at under 40 years (46% vs. 24%, *p*_trend_ = 0.034) (Figure 1C).

We also examined the differences among *BRCA1*/2 mutations in relation to the GSS score. Intriguingly, patients with *BRCA1* mutations had significantly higher median GSS scores compared to the *BRCA2* mutant patients (90 vs. 46, *p* = 0.018) (Figure 1C). The top recurrent mutation was *BRCA1* V409*, which was associated with the highest median GSS score of 97 (Figure 1D). It is noteworthy that the three Ashkenazi Jewish pathogenic founder mutations were not detected in our cohort. Taken together, our findings suggest a differential phenotypic impact of *BRCA1* mutations compared to *BRCA2* mutations.

Several reports have shown high concordance of in‐house HRD testing compared with central testing using Myriad MyChoice CDx [2, 4, 5]. The HRD rate in our cohort was similar to results from locally performed Myriad testing in a real‐world cohort [6]. However, the rate was relatively lower than what was reported in clinical trials, likely due to biomarker enrichment bias in these trials. To the best of our knowledge, this is the first report from limited resource settings aiming to bridge the gap in cancer care disparities. Locally performed HRD testing was found to be feasible and mitigated the high cost and lengthy turnaround time associated with central testing.

## Author Contributions

K.S.S., L.K. and H.A.A.: conceptualization and study design. H.A.A., L.K., B.G., E.B. and T.E.: data curation and patient recruitment. B.G.: project administration. K.S.S. and L.K.: formal analysis. K.S.S.: writing – original draft. All authors: writing – review and editing.

## Conflicts of Interest

K.S.S.: outside of this scope of work, has received research funding from Novartis Pharma AG Basel, Cellular Therapeutics Limited, InstillBio and Adaptimmune. L.K. received honoraria from Roche, AstraZeneca, Novartis, Jansen, Pfizer, Eva, Sandoz, Hikma and MSD. H.A.A. received honoraria from Roche, AstraZeneca, Novartis, Jansen, Pfizer, Eva, Sandoz, Hikma and MSD. All other authors have no conflicts of interest to declare.

## Data Availability

The data that support the findings of this study are available from the corresponding author upon reasonable request.
